# Association between the Dutch Obesogenic Built-environmental CharacterisTics (OBCT) index and 14-year cardiovascular disease incidence: a population-based cohort study of 4.4 million adults

**DOI:** 10.1016/j.lanepe.2025.101494

**Published:** 2025-10-15

**Authors:** Paul Meijer, Thao Minh Lam, Joline WJ. Beulens, Diederick E. Grobbee, Jeroen Lakerveld, Ilonca Vaartjes

**Affiliations:** a Julius Center for Health Sciences and Primary Care, University Medical Center Utrecht, Utrecht University, Utrecht, 3508 GA, the Netherlands; b Upstream Team, Amsterdam UMC, Vrije Universiteit Amsterdam, Amsterdam, 1105 AZ, the Netherlands; c Department of Epidemiology and Data Science, Amsterdam UMC, Location Vrije Universiteit Amsterdam, Amsterdam, 1105 AZ, the Netherlands; d Amsterdam Public Health, Health Behaviours & Chronic Diseases, Amsterdam, 1105 AZ, the Netherlands

## Abstract

**Background:**

The Dutch Obesogenic Built-environmental CharacterisTics (OBCT) index, which has been previously associated with higher BMI, waist circumference and increased prevalence of obesity, offers a composite measure of such exposures. This study investigated the association between OBCT index scores and 14-year CVD incidence in adults.

**Methods:**

This population-based cohort study included 4.4 million Dutch residents aged ≥40 years with no history of CVD as of January 1, 2006. Participants resided at the same address from January 1, 2004, to December 31, 2019, or until death. The OBCT index, ranging from 0 to 100, quantified the obesogenicity of participants' living environments at baseline. CVD outcomes included overall CVD incidence, coronary heart disease (CHD), stroke, and heart failure (HF). Cox proportional hazard models were used to estimate hazard ratios (HRs) and 95% confidence intervals (CIs).

**Findings:**

A 10-point higher OBCT index score was associated with a 1.1% (HR: 1.011; 95% CI: 1.009–1.013) higher risk of any CVD. Individuals in the highest quintile had a 4.9% (HR: 1.049; 95% CI: 1.038–1.060) higher risk of CVD compared to those in the lowest quintile, corresponding to 37 additional events per 10,000 persons at 10 years. A 10-point higher OBCT index was also associated with CVD mortality (HR: 1.010; 95% CI: 1.006–1.014) and for specific outcomes: CHD (1.1% higher risk), stroke (0.9% higher risk), and HF (1.2% higher risk). Associations were stronger in the highest income group and among individuals exposed to high air pollution levels.

**Interpretation:**

Living in a more obesogenic environment is associated with a higher 14-year risk of CVD. Although the effect sizes are modest at the individual level, their significance is amplified by the widespread nature of the exposures studied.

**Funding:**

This work is supported by EXPOSOME-NL. EXPOSOME-NL is a Dutch consortium funded through the Gravitation programme of the Dutch Ministry of Education, Culture, and Science and the Netherlands Organization for Scientific Research (NWO grant number 024.004.017).

**Research in context**

**Evidence before this study**

Obesogenic environments are defined by neighbourhood characteristics that encourage physical inactivity and unhealthy dietary habits. On April 1, 2024, we searched PubMed without language or time restrictions using the following strategy: ((“exposome”[MeSH] OR exposome[tiab] OR “built environment”[MeSH] OR “built environment”[tiab] OR (environment∗[tiab] AND (built[tiab] OR living[tiab] OR lifestyle[tiab] OR obesogenic[tiab] OR neighbo∗[tiab] OR food[tiab] OR physical activity[tiab])))) AND (“cardiovascular diseases”[MeSH] OR cardiovascular disease∗[tiab] OR cardiometabolic disease∗[tiab] OR heart disease∗[tiab] OR coronary artery disease∗[tiab] OR ischemic heart disease∗[tiab] OR myocardial infarction∗[tiab] OR stroke∗[tiab] OR cerebrovascular disease∗[tiab] OR heart failure[tiab]). We included studies of the general adult population if they examined objectively measured neighbourhood environmental exposures and reported associations between these factors and cardiovascular disease (CVD) outcomes. We also previously published an umbrella review on the built environment and CVD, as well as a systematic review on the food environment and CVD in 2023. Collectively, this demonstrated a growing body of evidence of a link between obesogenic environmental features, such as fast-food restaurants, greenspaces, and walkability, and CVD outcomes. However, most studies to date have been cross-sectional and examined single exposures. To advance the field, there is a need for large, population-based studies assessing multiple obesogenic exposures and tracking incident CVD longitudinally.

**Added value of this study**

Our research presents novel findings from a registry based nationwide cohort of over 4 million Dutch adults, followed over 14 years, showing that obesogenic environments are linked to a higher risk of CVD, including coronary heart disease (CHD), stroke and heart failure (HF). In our study, obesogenic environments were assessed using the Obesogenic Built-environmental CharacteriTics (OBCT) index that combines high resolution GIS data of the built environment, including food environment, physical activity infrastructure, and transportation, at residential address level. We observed that associations were stronger among individuals from higher-income groups and those exposed to high levels of air pollution (PM_2.5_), suggesting potential effect modification by socioeconomic and environmental vulnerability factors.

**Implications of all the available evidence**

Our findings provide robust, longitudinal evidence linking more obesogenic living environments to increased risk of CVD, reinforcing the role of the built environment in shaping public health. These results support the incorporation of environmental health determinants into urban planning, cardiovascular disease prevention strategies, and public health policy. Reducing the obesogenic potential of neighbourhoods by promoting active transport, and improving access to healthy foods, may contribute meaningfully to reducing the burden of CVD in Europe and beyond.

## Introduction

The impact of the built environment on cardiovascular health is increasingly recognised.^1^ Substantial evidence suggests that the structure of a neighbourhood, including its street design, green spaces, and accessibility to retail food and commercial areas, is associated with dietary behaviours and levels of physical activity.^2^, ^3^, ^4^ This interplay between neighbourhood design and lifestyle behaviours contributes to the concept of an “obesogenic environment”, wherein the surroundings may foster conditions conducive to obesity and related chronic diseases.^5^

A growing body of evidence supports the association between obesogenic environmental factors and cardiometabolic outcomes, including overweight and obesity, high cholesterol, type 2 diabetes, hypertension, and cardiovascular disease (CVD) events.^6^, ^7^, ^8^ Less walkable neighbourhoods are linked to a higher prevalence and/or higher risk of overweight and obesity,^2^^,^^9^ as well as higher rates of hypertension, arterial stiffness, and major CVD events.^10^ Exposure to greenspace is associated with lower blood pressure and prevalence of hypertension,^11^ and greater access to greenspace is associated with lower odds of overweight/obesity.^12^ The food environment, including supermarket availability and fast-food exposure, shows mixed associations with obesity.^13^ However, certain elements of the consumer and neighbourhood food environments could improve dietary intake and potentially impact BMI in some adult populations.^14^ A higher density of fast-food restaurants is most consistently associated with an increased occurrence of CVD events.^8^

However, most evidence comes from cross-sectional studies. Systematic reviews emphasise the need for longitudinal studies.^6^^,^^8^^,^^10^^,^^15^ Moreover, previous studies have mostly examined one exposure at a time, but this approach is limited because individuals are typically exposed to multiple environmental factors simultaneously.^6^^,^^7^^,^^16^ To reflect the complexity of real-world conditions, it is important to study clusters of exposures to gain a comprehensive understanding of their influence on health, especially since unhealthy environmental factors tend to cluster together.^17^

To capture co-occurrence of exposures, a composite indicator approach is increasingly used to reduce multicollinearity, avoid overadjustment; and to create actionable entry points for policy applications. Various composite indicators have been created to quantify multiple objectively measured built environment characteristics. Examples include the Heart Healthy Hoods (HHH) index for cardiovascular health in Madrid,^18^ the Obesogenic Built-environmental CharacterisTics (OBCT) index for obesity in the Netherlands,^16^ and the Childhood Obesogenic Environment Index (COEI) for childhood obesity in the USA.^19^

Studying the association between such indices and health outcomes has not been done often. In Madrid, the HHH index showed varied associations with CVD incidence depending on location,^18^ though the analysis was conducted at areal level rather than the individual level. In the USA, an adapted version of the COEI for adults was associated with higher odds of self-reported CVD.^20^ The Dutch OBCT index was significantly associated with a higher BMI and waist circumference, and a higher prevalence of overweight, obesity and hypertension.^21^^,^^22^ However, these studies are all cross-sectional.

To expand these observations, the purpose of this study was to investigate the association between exposure to a more obesogenic environment, as measured by the Dutch OBCT index, and 14-year CVD incidence in adults. We hypothesised that exposure to a more obesogenic built environments is associated with a higher 14-year incidence of overall CVD, CVD mortality, coronary heart disease (CHD), stroke, and heart failure (HF).

## Methods

### Study design and participants

We conducted a longitudinal cohort study using national registry data accessed through Statistics Netherlands (Centraal Bureau voor de Statistiek, CBS in Dutch). We combined four national registers: the Population Register,^23^ the Hospital Discharge Register,^24^ the National Cause of Death Register,^25^ and the system of social statistical datasets.^26^ The Population Register is based on municipal records which provide demographic information (e.g. date of birth, sex, and residential address) for every registered resident in the Netherlands. Since 2013, the administrative and medical data of hospital admissions are submitted by hospitals to the National Hospital Care Registration (Landelijke Basisregistratie Zorg (LBZ)). These data are managed by Dutch Hospital Data (DHD). DHD performs several category and relationship checks on the submitted data, after which hospitals can submit corrected data if necessary. After the final LBZ annual files have been established, they are delivered to CBS. For the registration years 1995–2012, hospital admissions were recorded in the administrative national medical registration (Landelijke Medische Registratie (LMR), the predecessor of the LBZ. Data were recorded by hospital administration at each admission. Upon discharge, the medical data were completed by or on behalf of the medical specialist on the discharge form. These data were then coded and registered in the LMR by the hospital's medical administration. The hospitals provided the LMR data to the registrar, who performed checks. Subsequently, CBS received the final annual LMR files from DHD. Hospital admission data include inpatient hospital care, day care, and observations (≥4 h). Admission causes are classified according to the 9th and 10th revision of the International Classification of Diseases (ICD-9 and ICD-10). ICD-9 was used for admissions up to and including 2013, and ICD-10 has been used for all admissions after 2013. The National Cause of Death Register receives information on cause and date of all deceased persons in the Netherlands from the legal reporting system. Up to 2012, for each death, a physician issues the death certificate including a cause of death according to ICD-10.^25^ From 2012 onwards, an automatic coding of mortality statistics was used.

The study period spanned from 1 January 2006 to 31 December 2019. Baseline was set on 1 January 2006, as this was the earliest year for which exposure data was available. Although hospitalisation and mortality data extend beyond 2019, we limited our follow-up to 31 December 2019, due to uncertainties around exposure, mobility patterns and health outcomes during the COVID-19 pandemic. All Dutch residents were included in the cohort if they were 40 years or older at baseline and had no recorded history of CVD, defined as no hospital admission for CVD between 1 January 1996, and 31 December 2005. The threshold of 40 years was chosen because the absolute risk of CVD events in the near term (e.g. 10 years) is generally low for younger adults. To be included, people were required to have lived at the same address from 1 January 2004 to 31 December 2019, or until death. This ensured that individuals had been living at the same address for two years before baseline to avoid the potential impact of a recent move. Individuals were considered to have moved if they changed address and did not change back to the same address for more than three months. We excluded residents if they had individual records indicating they lived at institutional addresses.

### Exposure: neighbourhood obesogenicity

**Fig. 1:**
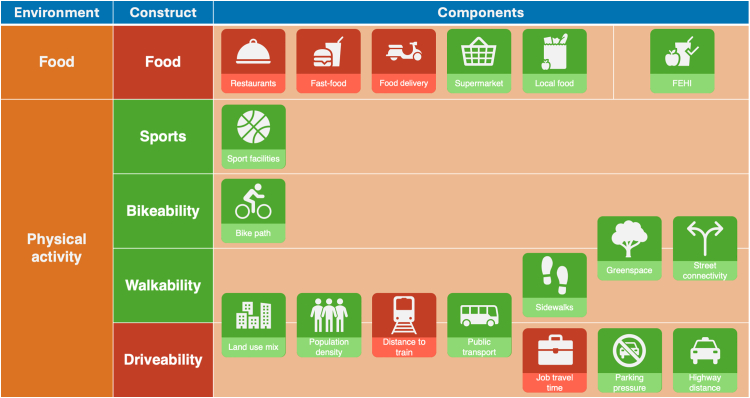
Conceptual framework for composing the Obesogenic Built environment CharacterisTics (OBCT) index. Red = obesogenic/unhealthy; green = leptogenic/healthy; FEHI = Food Environment Healthiness Index.

Neighbourhood obesogenicity was operationalised according to the Obesogenic Built-environmental CharacterisTics (OBCT) index. More information on the index development is published elsewhere.^16^ The OBCT index is a combination of the neighbourhood food- and physical activity environment, encompassing five constructs: food environment, sports facilities, bikeability, walkability, and driveability (Fig. 1).

The food environment was measured in two different variations: (1) the Food Environment Healthiness Index (FEHI), which combines the density, distance, and healthiness of food retailers; and (2) using the densities of five main food retailers. Sport facilities was defined as the point density of sports facilities including only sports which require physical activity (excluding chess, draughts, board games, shooting, bridge). Bikeability, walkability, and driveability are composite indicators of neighbourhood attributes facilitating cycling, walking, and car use respectively (Fig. 1). All geographical data for the OBCT index were centralised, operationalised, and provided by the Geoscience and Health Cohort Consortium (GECCO).^27^ A detailed description of each component, including data sources, and references can be found in Appendix 1 and Supplementary Table S1.

**Fig. 2:**
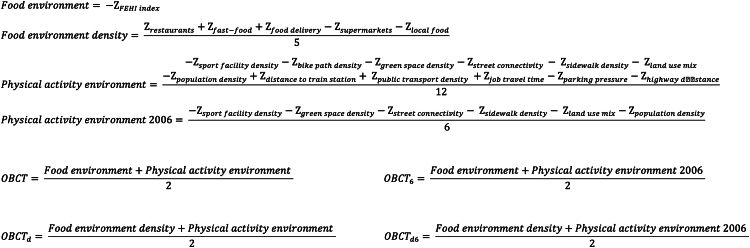
Equations for calculating the Obesogenic Built-environmental CharacterisTics (OBCT) index. OBCT_6_ = index using only the components available at or near baseline. OBCT_d_ = index using the densities of various food retailers instead of the Food Environment Healthiness Index (FEHI). OBCT_d6_ = index using only the components available at or near baseline, and the densities of various food retailers instead of the FEHI.

All components were z-standardised to have a median of zero and a mean absolute deviation of one to ensure a consistent scale. Median and mean absolute deviation were used instead of mean and standard deviation because most components were not normally distributed. To reduce the impact of outliers, we applied Winsorization to all components, adjusting the values to the 5th and 95th percentiles. We calculated the environmental scores by averaging the z-scores of all the components in each respective environment, considering their positive or negative contribution to obesogenicity (Fig. 2). The OBCT index was calculated by averaging the scores for the food and physical activity environment. The index was then normalised to range from 0 to 100, with a higher score indicating higher levels of obesogenicity. The index was calculated for circular buffers with a 1000-m radius around participants' home address. A 1000-m radius was chosen as individuals tend to perceive neighbourhoods to be smaller than the actual administrative neighbourhood, which extend beyond 1000 m.^28^ The index was also calculated for a smaller 500-m radius for sensitivity analysis.

### Index versions/sensitivity analysis

The OBCT index was originally developed for 2016. Since not all components were available for the baseline year of the study (2006), we calculated the index using only the components available at or near baseline (OBCT_6_) for comparison (Fig. 2). We calculated the OBCT index for our main analyses using the Food Environment Healthiness Index (FEHI) as measure of the obesogenic food environment, and we also calculated the OBCT index using the densities of various food retailers for comparison (OBCT_d_, OBCT_d6_).

### Outcome: incident CVD

Incident CVD was obtained from the Dutch Hospital Discharge Register and the National Cause of Death Register.^24^^,^^25^ We collected the primary and secondary causes of hospital admission, the date of hospital admission, as well as the cause and date of death. The ICD codes used for CVD are provided in Supplementary Table S2. Incidence of CVD was defined as the first hospital admission due to any CVD, or out of hospital death due to any CVD, whichever came first. We also assessed the incidence of specific CVD events including hospital admission or death due to CHD, stroke, or HF.

### Covariates

Individual- and neighbourhood level socio-demographic characteristics were obtained from CBS using the population register dataset (for residential address, biological sex, date of birth, migration background, partner status, household composition); the system of social statistical datasets—SSB (household income); and CBS geospatial data (neighbourhood urbanisation levels).^23^^,^^26^ Household income reflects the disposable income of household members standardised by the composition of the household (i.e. number of adults and children—by age) in Euros and was categorised into <25%, 25–75%, >75%. Partner status was categorised into single, married/registered partner, divorced/separated, or widowed. Migration background was based on the country of birth of the participants and of their parents (Native-Dutch, Other Western, Non-western). Comorbidities at baseline were determined using the Charlson Comorbidity Index (CCI) which was calculated using the Dutch Hospital Discharge Register data.^24^ Due to a high proportion of individuals without comorbidities, the score was dichotomised into comorbidities or no comorbidities.

Neighbourhood urbanisation level was defined by CBS in five categories according to the neighbourhood density of addresses per square kilometre: very high urbanisation (2500 address per km^2^ or more); high urbanisation (1500–2500 addresses per km^2^); moderate urbanisation (1000–1500 addresses per km^2^); low urbanisation (500–1000 addresses per km^2^); very low urbanisation (less than 500 addresses per km^2^). Area-level SES scores and air pollution data on annual average outdoor concentrations of particulate matter with diameters <2.5 μm (PM_2.5_) in μg/m^3^ at the residential address-level were obtained from GECCO. Objectively measured area-level SES score is a composite indicator consisting of neighbourhood-average education, income and position in the labour market.^29^^,^^30^ Higher scores indicate a higher area-level SES. Air pollution was derived based on a combination of model calculations and measurements from official measurement locations.^31^ Air pollution data with a resolution of 25 × 25 m were linked to all residential addresses in the Netherlands.

### Statistical analyses

We summarised the baseline characteristics as mean ± standard deviation (SD) or median and interquartile range (IQR) as appropriate for continuous variables, and number and percentage for categorical variables; for the total sample as well as stratified by quintiles of the OBCT index.

Due to the low proportion of cases dropped due to missing data (<5%) we conducted complete case analyses. We used Cox proportional hazards regression modelling to estimate the association between the OBCT index score and the incidence of CVD. Person-time for each individual was calculated from baseline until the first hospital admission due to any CVD, or out-of-hospital death due to any CVD, death due to other causes, or end-of-study date, whichever came first. We reported hazard ratios (HR) and 95% confidence intervals (CI) for each quintile of the OBCT index relative to the lowest quintile, and continuous per 10-point increase in the OBCT index score. All models were adjusted for age at baseline, biological sex, migration background, partner status, household income, area-level SES, comorbidities, PM_2.5_ concentration, and urbanicity. To assess the proportional hazards assumption, we used Schoenfeld residuals for continuous variables and log-minus-log plots for categorical variables. We did not observe any evidence for violations of the proportional hazards assumption.

We additionally used penalised spline regression with 3 degrees of freedom (DF) to visualise any potential non-linear dose-response relationship between the OBCT index score and CVD incidence.

We estimated absolute risk and corresponding 95% CI by calculating cumulative incidence at 10 years (1 − S(t)) from adjusted survival curves across quintiles of the OBCT index score using the adjustedCurves package in R with the direct adjustment method, which averages predicted survival probabilities over the covariate distribution of the study population.

We tested for effect modification using multiplicative interaction terms for age group (middle-aged (40–60 years) and older adults (≥60 years)), sex (males and females), household income (<25%, 25–75%, >75%), area-level SES (above and below average area-level SES score), urbanicity (<1000, 1000–2500, >2500 addresses/km^2^), comorbidities (yes/no), and PM_2.5_ (above and below median PM_2.5_ exposure). As sensitivity analysis, all exposures were analysed based on a 500 m Euclidian buffer radius. We also conducted Cox proportional hazards models using the different version of the index (Fig. 2) for comparison.

To examine the contributions of individual components and constructs to the overall OBCT index score, pairwise Spearman correlations between components were assessed and presented in a correlation matrix form.

We defined statistical significance as a p-value <0.05 (two-sided), and a false discovery rate correction (q-value) was applied for multiple testing. All statistical analyses were performed with R version 4.2.3 and R studio version 2023-06-2+561.

### Ethics approval

All data linkages and analyses were conducted in line with the policy from CBS and privacy legislation in the Netherlands. Ethical approval was not required for the present study.

### Role of funding source

This work is supported by EXPOSOME-NL. EXPOSOME-NL is a Dutch consortium funded through the Gravitation programme of the Dutch Ministry of Education, Culture, and Science and the Netherlands Organization for Scientific Research (NWO grant number 024.004.017). The funder played no role in the study design, data analysis, data interpretation, or the writing of this report.

## Results

**Fig. 3:**
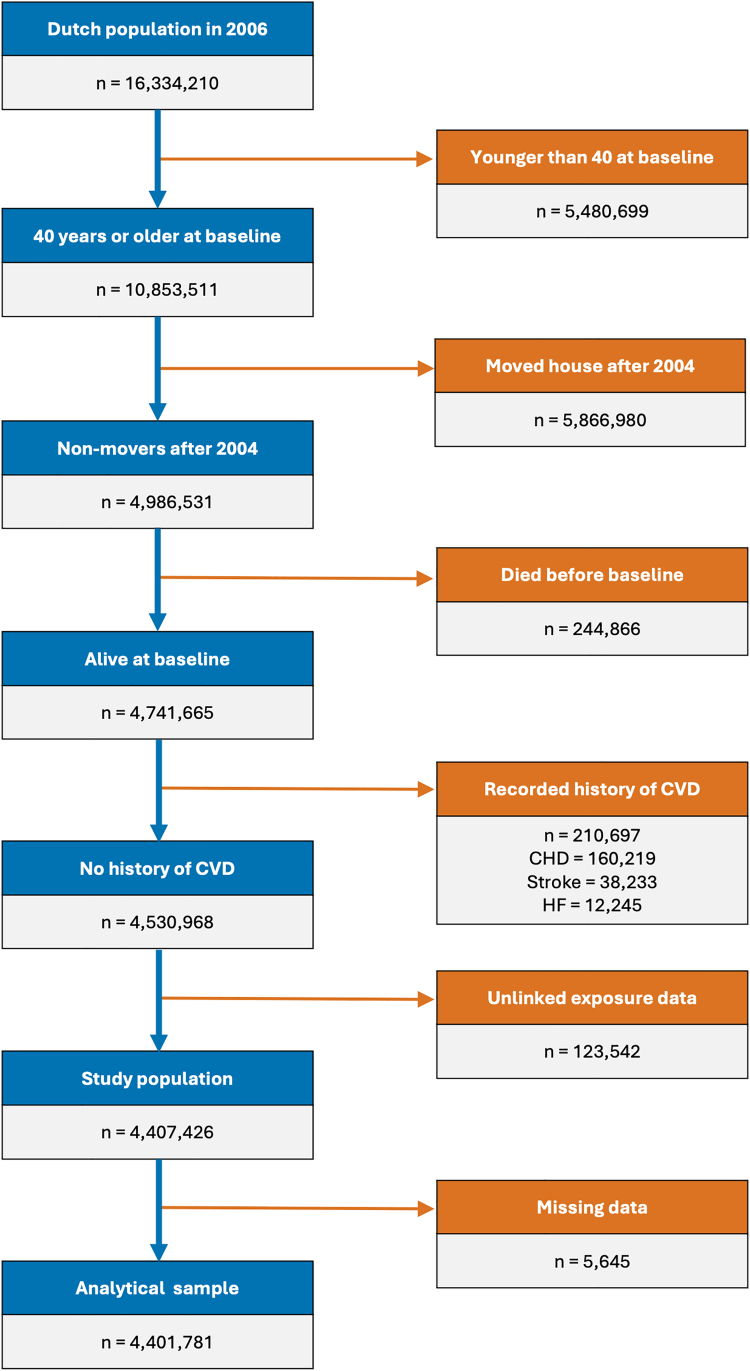
Flowchart of the study population.

Of 4,530,968 individuals who met the inclusion criteria, we additionally excluded 123,542 (2.7%) individuals who had unlinked exposure data, resulting in a study population of 4,407,426 individuals (Fig. 3). There were 5645 (0.1%) individuals dropped from the analyses due to one or more missing covariates. Individuals with missing data were more likely to have a migration background, be single or separated, and have a high income (>75%). Their exclusion did not alter the overall distribution of characteristics in the sample (Supplementary Table S3). The distribution of the baseline OBCT index for those included in the cohort (non-movers) and individuals excluded because they relocated within the Netherlands were highly similar (Supplementary Figures S1 and S2).

### Study sample characteristics

The median age at baseline of the study population was 56 (IQR: 48–65) years, 51.4% were female, 91.2% had no migration background, 74.1% were living with a partner, and 62.3% lived in a neighbourhood that was not urbanised to moderately urbanised (Table 1). Median annual disposable household income was €19,790.

**Table 1:** Population characteristics of the total study sample as well as stratified by the Obesogenic Built-environmental CharacterisTics (OBCT) index quintiles.

	All	First quintile (<39)	Second quintile (39–46)	Third quintile (46–52)	Fourth quintile (52–62)	Fifth quintile (>62)
n	4,407,426	881,486	881,485	881,485	881,485	881,485
**Follow up (median [IQR])**	14.0 [14.0; 14.0]	14.0 [14.0; 14.0]	14.0 [14.0; 14.0]	14.0 [14.0; 14.0]	14.0 [14.0; 14.0]	14.0 [14.0; 14.0]
**Sex (%)**						
Female	51.4	52.8	51.5	51.1	51.0	50.5
**Age at baseline (median [IQR])**	56 [48; 65]	56 [48; 66]	56 [48; 65]	55 [47; 65]	56 [48; 65]	56 [48; 65]
**Migration background (%)**						
None	91.2	86.6	89.8	91.6	93.0	94.9
Other Western	4.4	5.7	4.7	4.3	4.0	3.3
Non-western	4.5	7.7	5.5	4.2	3.1	1.8
**Partner status (%)**						
Single	9.7	11.6	10.1	9.7	8.8	8.5
Widowed	8.9	9.5	8.7	8.6	8.8	9.2
Separated	8.2	10.4	8.4	8.1	7.5	6.6
Partner^a^	73.1	68.4	72.8	73.6	75.0	75.7
**Annual household income in € (%)**						
<25% (<16,039)	24.3	25.0	23.7	24.0	24.0	24.7
25–75% (16,039–27,630)	50.4	49.1	49.1	50.2	51.5	51.9
>75% (>27,630)	25.3	25.8	27.1	25.7	24.5	23.3
NA	0.1	0.1	0.1	0.1	0.1	0.1
**Comorbidities (%)**	5.1	5.3	5.0	5.0	5.0	5.1
**Area-level SES**^b^**(mean (SD))**	0.21 (0.81)	−0.01 (1.00)	0.21 (0.86)	0.23 (0.77)	0.27 (0.69)	0.35 (0.62)
**Urbanisation (%)**						
<500	21.4	3.2	17.7	18.7	22.3	45.3
500–1000	20.8	7.8	16.3	22.1	30.0	27.8
1000–1500	20.1	20.0	21.6	22.4	21.8	14.5
1500–2500	23.6	38.6	27.0	23.2	19.0	10.2
>2500	14.1	30.3	17.5	13.6	6.9	2.3
**PM**_**2.5**_**in μg/m^3^ (median [IQR])**	15.2 [14.0; 15.8]	15.5 [14.3; 16.1]	15.2 [13.9; 15.9]	15.1 [13.8; 15.8]	15.0 [13.9; 15.8]	15.0 [13.7; 15.7]

a Partner included both marriage and registered partnership.

b Socio-economic status.

Those living in neighbourhoods with the highest OBCT index scores were more often male, had no migration background, and lived with a partner (Table 1). Neighbourhoods with a higher OBCT index score had a higher mean area-level SES and were less likely to be urbanised. There was also a slight decrease in mean PM_2.5_ exposure with higher OBCT index quintiles.

### CVD incidence

**Fig. 4:**
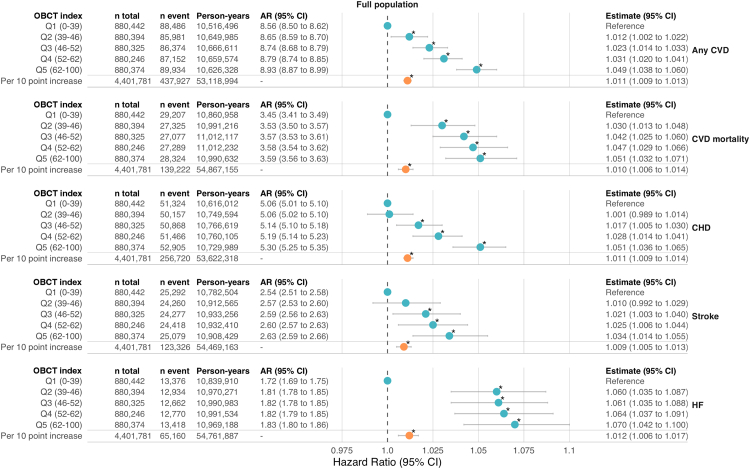
Associations between the Obesogenic Built-environmental CharacterisTics (OBCT) index score and cardiovascular outcomes. Models adjusted for age, sex, migration background, partner status, household income, comorbidities, area level SES, PM_2.5_ exposure, and urbanicity. ^∗^Remained statistically significant after false very rate correction for multiple testing. CVD = cardiovascular disease; CHD = coronary heart disease; HF = heart failure; AR = absolute risk at 10 years.

During 53,118,994 person-years of follow-up, 437,927 (9.9%) individuals were hospitalised due to CVD, of which 256,720 for CHD (5.8%), 123,326 for stroke (2.8%), and 65,160 for HF (1.5%). There were 139,222 (3.2%) deaths due to CVD. We observed that a higher OBCT-index score was associated with a higher risk of any CVD (Fig. 4). Specifically, each 10-point higher OBCT-index score (range: 0–100) was associated with a 1.1% (HR: 1.011; 95% CI: 1.009–1.013) higher risk of any CVD. Individuals in the highest quintile (Q5) had a 4.9% (HR: 1.049; 95% CI: 1.038–1.060) higher risk of CVD compared to those in the lowest quintile (Q1). The hazard ratio increased progressively across quintiles, indicating a dose-response relationship, also observed in the penalised spline regression model (Supplementary Figure S3). At 10 years, the cumulative incidence of any CVD was 8.93% in the highest quintile versus 8.56% in the lowest quintile, indicating approximately 37 additional events per 10,000 persons attributable to living in the most obesogenic environments.

There were consistent associations found between a 10-point higher OBCT-index scores and increased risks of specific cardiovascular outcomes (Fig. 4). For CHD, individuals in the highest exposure quintile (Q5) had a 5.1% (HR: 1.051; 95% CI: 1.036–1.065) higher risk compared to those in the lowest quintile (Q1) with approximately 24 additional events per 10,000 persons attributable to living in the most obesogenic environments.

Similar trends were observed for stroke and HF, with the highest quintile showing significantly elevated risks and attributable events. The highest exposure quintile (Q5) was associated with a 3.4% (HR: 1.034; 95% CI: 1.014–1.055) higher risk of stroke with 9 additional events per 10,000 persons, and a 7.0% (HR: 1.070; 95% CI: 1.042–1.100) higher risk of heart failure with 11 additional events per 10,000 persons.

We also observed that each 10-point higher OBCT-index score was associated with a 1.0% (HR: 1.010; 95% CI: 1.006–1.014) higher risk of CVD mortality (Fig. 4). Individuals in the highest quintile (Q5) had a 5.1% (HR: 1.051; 95% CI: 1.032–1.071) higher risk of CVD mortality compared to those in the lowest quintile (Q1). This corresponds to 14 additional deaths due to CVD per 10,000 persons at 10 years.

We observed comparable results when using a 500 m buffer instead of a 1000 m buffer, although the HRs were slightly attenuated (Supplementary Figure S7). Both the 2006 OBCT index and the full OBCT index yielded similar associations (Supplementary Figures S3 and S4). Additionally, utilising the densities of various food retailers instead of the FEHI index produced similar results, with a slight attenuation in the HRs (Supplementary Figures S5 and S6).

### Effect modification

**Fig. 5:**
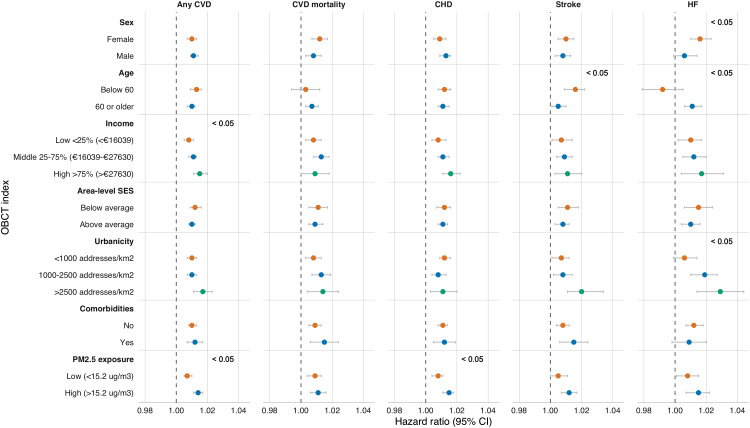
Associations between a 10-point increase in the Obesogenic Built-environmental CharacterisTics (OBCT) index and cardiovascular outcomes, stratified by age, sex, income, area-level SES, Urbanicity, comorbidities, and PM_2.5_ exposure. CVD = cardiovascular disease; CHD = coronary heart disease; HF = heart. <0.05 indicates significant interaction term.

For any CVD, a significant interaction was found between standardised household income groups and the OBCT index (Fig. 5). Each 10-point increase in the OBCT index score was associated with a higher risk of CVD across all income groups, with the highest HR observed in the top 25% income group and the lowest HR in the bottom 25% income group. Additionally, we observed effect modification by PM_2.5_ exposure (Fig. 5). The association was significantly stronger in the high PM_2.5_ exposure group (≥15.2 μg/m^3^) compared to the low exposure group (<15.2 μg/m^3^). This difference of PM_2.5_ was also seen for CHD specifically.

There was a significant interaction between age groups and the OBCT index for stroke and HF (Fig. 5). Adults younger than 60 had a stronger association between OBCT index scores and stroke compared to those who were 60 or older. Conversely, the association between OBCT index scores and HF was stronger in adults 60 or older. Lastly, for HF, the associations were stronger in females compared to males, and in urban areas (>2500 addresses/km^2^) compared to rural areas (<1000 addresses/km^2^) (Fig. 5).

### Spearman correlation

The OBCT index was most strongly correlated with the FEHI index (r = −0.76) (Supplementary Figure S8). Correlations between the OBCT index and specific food outlet densities were modest and generally negative. By contrast, the PA environment score (higher score equals unfavourable PA environment) was moderately positively correlated with the OBCT index (r = 0.56). Several PA-related built environment features showed notable negative correlations with the OBCT index, including: bike pathway density (r = −0.44), sidewalk density (r = −0.46), street connectivity (r = −0.40), population density (r = −0.46), and public transport density (r = −0.37). Greenspace and sports facilities were to a lesser extend negatively correlated with the OBCT index.

## Discussion

In this large nationwide cohort study, exposure to a more obesogenic environment (measured by the Dutch OBCT index) was significantly associated with a higher 14-year risk of CVD. The relationship between a higher OBCT-index score and CVD risk was approximately linear, with every 10-point increase in the index corresponding with a 1.1% higher risk. This association held not only for overall CVD but also extended to the specific outcomes CVD mortality, CHD, stroke, and HF, all showing associations of similar magnitude. Our findings remained robust across various sensitivity analyses, including varying buffer size and index components. Subgroup analyses showed a higher risk in the highest compared to the lowest income group, and in individuals with high compared to low PM_2.5_ exposure. While the absolute risk increase associated with exposure to the highest versus lowest quintile of obesogenic environment score was modest at 37 events per 10,000 persons over 10 years, this translates into meaningful impacts when considered at the population level assuming a causal relationship. For example, in a city of 500,000 residents, if 20% live in areas with the highest obesogenic scores, this excess risk could result in approximately 370 additional events over a decade. When extrapolated to the scale of the entire country, the public health burden becomes more substantial, potentially accounting for thousands of additional events.

Our correlation analyses show that the OBCT index is more strongly linked with overall food environment quality than with individual food outlet densities, highlighting the importance of the broader, composite context. Previous research in the Netherlands indicates that the overall healthiness of the food environment is more strongly related to CVD risk than specific types of food outlets.^32^ In the physical activity environment, we observed that better infrastructure for active transport and improved connectivity were linked to less obesogenic environments, whereas greenspace and sports facilities contributed less. Earlier work showed that low walkability in the Netherlands is associated with higher CVD risk,^33^ further underscoring the role of active transport infrastructure.

In response to the consistent call for longitudinal studies, our study, to our knowledge, is the first to study the relationship between a composite (obesogenic) built environment index and CVD longitudinally. Our findings are consistent with two previous cross-sectional studies. Guo et al. adapted the American Childhood Obesogenic Environment Index (COEI) measured at county level (ranging from 0.026 to 376,869 km^2^ in size) to reflect obesogenic environments for adults.^20^ They found that older adults living in counties with higher COEI scores had 7.4% higher odds of self-reported CVD. In Madrid, Cebrecos et al. developed the multicomponent Heart Healthy Hoods Index (HHH).^18^ They observed that at census section area-level (on average 0.2 km^2^ [0.007–94.7 km^2^]) heart-healthy urban environments were spatially associated with a lower age-adjusted CVD prevalence rate. Our study advances these insights by using large-scale individual-level data and by evaluating obesogenic environments within a 1000-m radius around residential addresses. This method provides a more precise reflection of each individual's immediate neighbourhood conditions compared to varying geographic units like counties or census sections.

Some studies have examined composite indices in relation to cardiometabolic risk factors. In previous work, we examined the association of the Dutch OBCT index with cardiometabolic risk factors cross-sectionally.^21^ In a pooled analysis of five Dutch population-based cohort studies, we found significant associations between a higher index score and higher BMI, as well as a higher prevalence of overweight, obesity, and hypertension. Moreover, the OBCT index was also cross-sectionally associated with waist circumference.^22^ Other studies of composite indicators have also observed associations with cardiometabolic risk factors. For instance, Guo et al. found that older adults living in obesogenic environments had greater physiological dysregulation, determined using indicators such as systolic blood pressure, diastolic blood pressure, total cholesterol, HDL-cholesterol, and BMI.^20^ The current data support and extends these findings by demonstrating that the association between obesogenic environments and cardiovascular health risks extends beyond intermediate risk factors to downstream CVD outcomes.

The current and previous findings fit within a broader theoretical framework that connects the obesogenic built environment to CVD through two major pathways: lifestyle behaviours and environmental stressors.^34^ The link between lifestyle behaviours, such as diet and physical activity, and cardiometabolic health is well established.^35^ Increasing evidence suggests that the built environment is associated with these behaviours, with aspects like access to healthy foods and opportunities for physical activity playing critical roles.^2^, ^3^, ^4^ Several previous studies show that features of the neighbourhood food environment, such as the availability and accessibility of fast food restaurants, and convenience stores are associated with lower diet quality.^4^^,^^14^ Previous work in the Netherlands has also shown that more walkable environments, captured by the walkability-related components included in the OBCT index, are associated with increased time spent walking.^36^ Given these pathways, the overall neighbourhood environment should logically affect CVD risk over time, a hypothesis supported by our findings. However, studies directly examining if and how lifestyle behaviours mediate this relationship are still limited. Adherence to physical activity guidelines, and dietary behaviour mediated 13.3% of the association between the OBCT index and waist circumference, though this finding did not reach statistical significance.^22^ Guo et al. found that behaviours such as diet, smoking, and alcohol consumption accounted for only about 3% of the cross-sectional association between obesogenic environments and CVD.^20^ They also noted that these behaviours and cardiometabolic risk factors might act through separate pathways. We did not have data on physical activity and dietary habits, so we could not test mediation effects. More research, preferably longitudinal, is needed to explore mediation by lifestyle behaviours and further unravel these mechanisms.

The second main pathway through which the built environment influences cardiovascular health involves harmful exposures and stressors such as air pollution and noise.^34^ Urban environments characterised by high traffic and high population density often have elevated levels of air pollution and noise, which have been linked to various health risks, including CVD.^15^ These environmental stressors often coincide with obesogenic exposures, such as higher density of fast-food outlets, potentially creating a compounded risk environment.^34^ Our study provides evidence of this compounded risk, particularly in areas with high PM_2.5_ exposure. This suggests that the combination of poor air quality and an obesogenic environment may exacerbate cardiovascular risk. The same could be true for other stressors such as noise. However, the opposite is also possible. Urban areas generally offer higher availability of public transport, green spaces, and active transport infrastructure, which are supportive for cardiovascular health.

We also observed a stronger association among individuals in the highest income group compared to those in the lowest. Individual with higher income may have more access to private transportation (e.g. cars), making them more likely to choose passive transportation in an environment that does not facilitate active travel. Individuals with higher incomes may also have more disposable income to spend on convenience foods, and dining out, when those options are present in the environment. Moreover, individuals with higher income levels are more likely to engage in sedentary occupations, such as desk work, which is independently associated with increased metabolic risk factors for CVD.^37^ We did not have data on lifestyle, and education and occupation to further explore these relationships, but our findings suggest that these factors warrant closer examination in future research.

The reason for other observed interactions remains unclear, but may be partly explained by differences in risk factor profiles across subgroups. Obesity is a stronger risk factor for HF, especially with preserved ejection fraction (HFpEF), in women, which might contribute to the stronger association of the OBCT index with HF in women.^38^ Similarly, the association between the OBCT index and stroke in adults younger than 60 could reflect the greater impact of obesity in this group.^39^ The stronger link between the OBCT index and HF in adults aged 60 and older seems counterintuitive, since risk factors such as obesity, hypertension, and type 2 diabetes confer a greater relative risk of HF in younger adults.^40^ Older adults generally have a higher baseline risk of HF due to age-related changes in cardiovascular structure and function, and comorbidities.^40^ Therefore, even a modest additional burden from an obesogenic environment might significantly amplify HF risk.

The relationship between the obesogenic built environment and health is complex, likely involving numerous feedback loops and interactions. Assessing individual exposures is challenging, as the true effect might be diluted by the presence of other correlated exposures or by the complexities of the system. More proximal relationships are also easier to test than more distal relationships. Given this complexity, our use of a composite index rather than individual exposures offers a more holistic assessment of the obesogenic environment and its association with CVD risk. In practice, the OBCT index could serve as a heatmap to highlight disparities in obesogenic exposures among different areas or populations. This can enable municipal and regional policymakers to identify specific environmental elements contributing to the local obesogenic score and tailor interventions accordingly.

The strengths of this study include a longitudinal design using a large nationwide cohort over a long period of follow-up and individual level registry data, allowing us to examine the temporal relationship between the environment and CVD extensively. Furthermore, the obesogenic built environment was quantified using a high-resolution, objectively measured geospatial index that uniquely combined the food and the physical activity environment.

However, our findings should be interpreted within the context of certain limitations. First, while we considered a broad range of individual and neighbourhood level covariates, we cannot rule out the possibility of residual confounding, particularly from risk factors such as smoking and alcohol use, for which data were not available in this study. For this reason we could also not investigate potential meditation by diet or physical activity, or BMI. Second, we focused on the obesogenicity of residential settings, disregarding various places where individuals spend a significant amount of their time, including workplaces, shopping districts, and recreational areas. Third, we assessed the obesogenic environment only at baseline. The built environment is dynamic, with significant changes in aspects like the food environment over time.^41^ Future research should incorporate methods and data that can better capture dynamic environmental exposures. Fourth, healthy individuals may choose their residential neighbourhood based on physical activity and/or food availability preferences. This residential self-selection could have led to overestimation of the relation between the obesogenic environment and CVD. We aimed to reduce the immediate impact of self-selection bias by focusing on non-movers, excluding individuals who moved close to baseline and potentially chose their residence based on factors that may be associated with both obesogenic environment features and health outcomes. Fifth, the use of a composite index may obscure the relative contributions of individual environmental components to CVD risk. This trade-off is inherent in the use of comprehensive indices, and future research should aim to unpack the roles of specific components. Nonetheless, we believe that the composite approach remains valuable for capturing the combined association of multiple built environment exposures with health. Sixth, while we focused on major cardiovascular outcomes with the highest public health burden, the obesogenic environment may show different associations with specific cardiovascular conditions. Future studies should investigate associations between the OBCT index and specific obesity-related cardiovascular conditions, such as cardiac valve disease, arrhythmias, and venous thromboembolism since these may reveal additional pathways linking the built environment to cardiovascular risk. Last, while our study is focused on the Netherlands, which benefits from high-quality health- and environmental data, the generalisability of our findings to other contexts may be limited. Other countries and regions have varying built environments, healthcare systems, and population characteristics, which could influence the applicability of our results elsewhere.

Living in a more obesogenic environment is associated with a higher 14-year risk of overall and specific CVD events. If this association is causal, at 10 years, 37 additional events per 10,000 persons attributable to living in the most obesogenic environments. The broader interplay between food environment healthiness and infrastructure for active transport and connectivity shapes the obesogenic environment. Considering the significant burden of CVD on public health, these findings provide support for strategies targeting obesogenic environments as part of a comprehensive approach to preventing cardiovascular disease at the population level.

## Contributors

PM, TML, JWJB, JL and IV conceived and designed the study. PM conducted data preparation, data analysis, data interpretation, and wrote the initial draft of the paper. TML, JWJB, DEG, JL and IV contributed to data interpretation and revision of the manuscript. All authors reviewed, edited and approved the final version of the paper. IV is the guarantor. The corresponding author attests that all listed authors meet authorship criteria and that no others meeting the criteria have been omitted.

The lead author affirms that the manuscript is an honest, accurate, and transparent account of the study being reported; that no important aspects of the study have been omitted; and that any discrepancies from the study as planned (and, if relevant, registered) have been explained.

## Data sharing statement

We used non-public microdata from Statistics Netherlands. Under certain conditions, the underlying encrypted microdata are accessible for statistical and scientific research. For further information contact microdata@cbs.nl. If verification of the analyses is desired and Statistics Netherlands provides access to the microdata, we will provide the R-scripts for cohort-building and analyses upon request to the corresponding author. The geodata can be requested from the Geoscience and Health Cohort Consortium (www.gecco.nl).

## Declaration of interests

All authors have completed the ICMJE uniform disclosure form at www.icmje.org/disclosure-of-interest/ and declare: PM and JWJB report support for the submitted work from NWO Gravitation grant Exposome-NL under grant No. 024004017.
