# Supplementary material for: Association between the Dutch Obesogenic Built-environmental CharacterisTics (OBCT) index and 14-year cardiovascular disease incidence: a population-based cohort study of 4.4 million adults
Source: Lancet Reg Health Eur. 2025 Oct 15;59:101494. doi: 10.1016/j.lanepe.2025.101494 (PMC12550154; doi:10.1016/j.lanepe.2025.101494)
Supplement: Supplementary Material [file mmc1.docx]

**Obesogenic environments and cardiovascular disease incidence: A population-based cohort study of 4.4M adults**

Paul Meijer,^a,b^ Thao Minh Lam,^b,c,d^ Joline WJ Beulens,^a,b,c,d^ Diederick E Grobbee,^a^ Jeroen Lakerveld,^b,c,d,*^ Ilonca Vaartjes^a,*^

^a^ Julius Center for Health Sciences and Primary Care, University Medical Center Utrecht, Utrecht University, 3508 GA, Utrecht, Netherlands

^b^ Upstream Team, Amsterdam UMC, Vrije Universiteit Amsterdam, 1105 AZ, Amsterdam, Netherlands

^c^ Amsterdam UMC, location Vrije Universiteit Amsterdam, Department of Epidemiology and Data Science, 1105 AZ, Amsterdam, Netherlands

^d^ Amsterdam Public Health, Health Behaviours & Chronic Diseases, 1105 AZ, Amsterdam, Netherlands

| **Table of contents:** | Page |
| --- | --- |
|  |  |
| Appendix 1: description of OBCT index components | 2 |
| Supplementary Table 1. Summary of components included in the OBCT index: operationalisation, source, and year of data collection | 6 |
| Supplementary Table 2. ICD codes used for cardiovascular diseases | 7 |
| Supplementary table 3. Population characteristics of the total study sample as well as stratified by individuals with and without missing data | 8 |
| Supplementary figure 1. Distribution of the OBCT index score at baseline among individuals included in the study. | 9 |
| Supplementary figure 2. Distribution of the OBCT index score at baseline among individuals excluded from the study because they moved to a different address within the Netherlands during follow-up. | 10 |
| Supplementary figure 3. Associations between the OBCT index score using only the components available at or near baseline, and any CVD using penalized spline regression (df = 3). | 11 |
| Supplementary figure 4. Associations between the full OBCT index score and any CVD using penalized spline regression (df = 3). | 12 |
| Supplementary figure 5. Associations between the full OBCT index score using the densities of various food retailers instead of the FEHI, and any CVD using penalized spline regression (df = 3). | 13 |
| Supplementary figure 6. Associations between the full OBCT index score using only the components available at or near baseline, and the densities of various food retailers instead of the FEHI, and any CVD using penalized spline regression (df = 3). | 14 |
| Supplementary figure 7. Associations between the Obesogenic Built-environmental CharacterisTics (OBCT) index in a 500m circular buffer score and cardiovascular outcomes. | 15 |
| Supplementary figure 8. Spearman correlation matrix of the OBCT index constructs and components. | 16 |

**Appendix 1: description of OBCT index components**

1. **Food environment**
   1. **Food Environment Healthiness Index (FEHI)**

Food retailer source data came from Locatus, a Dutch commercial company that collects retail outlet data. Locatus staff gather xy coordinates and type of retailers through field audits. Shopping areas are audited yearly, and non-shopping areas every 2-3 years. Ground truthing showed a ‘good’ to ‘excellent’ agreement with a positive predictive value of 0.90 for both location and classification of food retailers.^1^ For this study, the neighbourhood food environment at baseline (2006) was operationalised using the Food Environment Healthiness Index (FEHI), which combines the density, distance, and healthiness of food retailers.^2^ The FEHI provides a nuanced indication of the healthiness of the neighbourhood food environment based on the overall number of retailers selling food. This index was developed through a Delphi study where 20 Dutch scientists working in the field of nutrition and food environment rated a range of food retailers according to the profile of foods that they offered. Food retailer scores could range from -5 (less healthy food retailers) to +5 (healthier food retailers). In several anonymous rounds, scores and rationales for each outlet were provided, shared among the scientists and discussed until consensus was reached. An overview of included food retailers and their respective scores can be found elsewhere.^3^ The FEHI exposures were created by calculating grid-based kernel density distances based information on the food environment around each possible location (each 25x25 meters grid cel in a regular spaced raster) in a circle with a radius of 1000 meters. For each grid cell the distance weighted retailers per aggregation class (counts) were summed up and subsequently averaged over the corresponding circular neighbourhoods. If an individual had no food retailers present within 1000 metres around the home (<1%), a value of zero was assigned to them.

- 1. **Density of food retailers**

As alternative food environment exposure, we used the point location of each food retailer across the Netherlands at baseline (2006) to calculate the densities of separate food retail categories: food delivery outlets, fast-food outlets, restaurants, local food shops, supermarkets and convenience stores. The Kernel Density method was used to calculate grid-based kernel density distances to food retailers in a 1000-meter radius using a 25-metre grid cell size. The calculated value was linked to residential addresses under the corresponding grid cell.

1. **Physical activity environment**
   1. **Walkability**

Walkability is a validated seven-component index related to walking specifically and active transport in general.^4^ Population density was defined as the number of inhabitants per hectare. Densely populated areas encourage walking over driving due to accessibility of utilitarian destinations^5^ and avoidance of traffic congestion.^6^ Gridded population density data were obtained from Statistics Netherlands (CBS Statline) for the year 2006. Land use mix was measured using the land use entropy index, which captures how evenly land uses classes are distributed. The values range from 0 to 1 with 1 representing perfect mix of all relevant land uses.^7^ A mixed land use is theorized to enhance walking since more desirable destinations are close by.^8,9^ For this analysis, we obtained Dutch land use dataset for 2006 from the National Georegister and included the following five land use categories in calculating land use mix: 1) commercial (retail and catering); 2) socio-cultural services (school, universities, hospitals and medical services, museums and concert halls); 3) residential areas; 4) offices and public services; and 5) green space and recreation (parks and recreation areas, sports and leisure activity areas). These land use classes have been identified to be relevant for walking.^10^ Street connectivity was defined as the point density of true intersections (i.e., three or more legs) on road segments that are accessible for pedestrians (e.g., excluding highways). A higher density of intersections is thought to be correlated with more walking through increasing the number of choices for getting to a destination on foot.^11^ Intersections were derived from topographical maps of the Netherlands (TOP10 NL) for the year 2003. These data were obtained from data service of ESRI the Netherlands. Green space density was defined as the proportion of land devoted to parks, public gardens, forests and graveyards. Green space is sometimes classified as part of the natural environment rather than built, however, in urban settings green structures are more often planned and built as well. Green space data were obtained from the Dutch land use dataset 2006. Sidewalk density was defined as the area proportion of sidewalk. The rationale to include sidewalks as part of walkability is that it represents the availability of dedicated walking space, thus the safety aspect of walking for both transport and leisure purposes. Sidewalk data were obtained from the 2008 topographic map via ESRI the Netherlands. Public transport density was defined as the point density of all trams, buses, metros and ferries for short-range transport combined with density of train stations for long distance transport in 2018. Recent evidence showed that it promotes high frequency and longer distance of active transport modes, thereby increasing overall walking and also cycling in the Dutch context.^12-14^ Public transport data were obtained from Geographic service of the University of Groningen (Geodienst Rijksuniversiteit Groningen, Groningen, the Netherlands).

- 1. **Bikeability**

Bikeability is represented by one built-environmental component related to facilitate biking, which is bicycle pathway density. This was defined as proportion of the neighbourhood area that is dedicated to bicycle paths. This component was derived from the Dutch basic topographic map (Basisregistratie Topografie) TOP10NL for the year 2019. Other components potentially related to biking were already included in the walkability constructs: green space, retail and service density and street connectivity.

- 1. **Driveability**

Driveability is represented by three built-environmental component related to facilitate car use which are parking pressure, job density, and distance to the nearest highway.

Parking pressure, expressed as the ratio between parking capacity (spots available) and household car ownership within neighbourhoods in 2016. Parking space polygons were extracted from two different sources: the Dutch large-scale topographic map (Basisregistratie Grootschalig Topografie) for 2019, and the Dutch basic topographic map (Basisregistratie Topografie) TOP10 for 2019. Car ownership per household was retrieved from Statistics Netherlands at 5-digit postal code (PC5) level and aggregated to neighbourhood level. A second variable for demand management was constructed to capture paid parking using data from TO10 and the Netherlands Vehicle Authority. We thereby assessed the proportion of paid parking areas as opposed to the total parking in a neighbourhood. This implies that with more paid parking areas, residents are likely to have to obtain a parking permit, making the area somewhat less attractive to own a car as opposed to free parking in the neighbourhood.

Average time travel by car to 10,000 jobs in seconds was calculated as a measure of job density. This component was calculated by SPINlab from the Free University Amsterdam using 2018 paid work location data from the National Information System of Workplaces (LISA).

Distance to the nearest highway was defined as the distance from neighbourhood centroid to the nearest highway ramp for 2016 was readily available in the district and neighbourhood dataset (wijk- en buurtkaarten 2016) from Statistics Netherlands. Highway was defined as either an A- (national) or N- (provincial) highway, with distance measured in kilometres from neighbourhood centroid via road network to the nearest highway entry/exit.

Other components potentially related to driving were already included in the walkability construct: population density, land use mix, density of public transport stops and distance to the nearest train station.

- 1. **Sports facilities**

Density of sport facilities was calculated per km2 per postcode 4 area. Only sport facilities that are present in the Real Estate Monitor 2015 database in 2006 have been selected. These are: athletic tracks, indoor sport halls/ gyms, swimming pools (inside and outside), ice rinks, and artificial ski slopes.

**References**

1. Canalia C, Pinho MGM, Lakerveld J, Mackenbach JD. Field Validation of Commercially Available Food Retailer Data in the Netherlands. *Int J Environ Res Public Health* 2020;**17**. doi: 10.3390/ijerph17061946

2. Timmermans J, Dijkstra C, Kamphuis C*, et al.* 'Obesogenic' School Food Environments? An Urban Case Study in The Netherlands. *Int J Environ Res Public Health* 2018;**15**. doi: 10.3390/ijerph15040619

3. Wagtendonk A. Locatus 2016 – food environment per neighborhood. https://www.gecco.nl/app/download/8104243664/Metadatasheet%20-%20Neighborhood%20food%20environment%20Locatus%202016.pdf?t=1680264414 (August 29 2024)

4. Lam TM, Wang Z, Vaartjes I*, et al.* Development of an objectively measured walkability index for the Netherlands. *International Journal of Behavioral Nutrition and Physical Activity* 2022;**19**:50. doi: https://doi.org/10.1186/s12966-022-01270-8

5. Brown BB, Yamada I, Smith KR*, et al.* Mixed land use and walkability: Variations in land use measures and relationships with BMI, overweight, and obesity. *Health & Place* 2009;**15**:1130-1141. doi: https://doi.org/10.1016/j.healthplace.2009.06.008

6. Koska T, Rudolph F. The role of walking and cycling in reducing congestion: a portfolio of measures. 2017. doi:

7. Frank LD, Schmid TL, Sallis JF, Chapman J, Saelens BE. Linking objectively measured physical activity with objectively measured urban form: Findings from SMARTRAQ. *American Journal of Preventive Medicine* 2005;**28**:117-125. doi: https://doi.org/10.1016/j.amepre.2004.11.001

8. Feng J, Glass TA, Curriero FC, Stewart WF, Schwartz BS. The built environment and obesity: A systematic review of the epidemiologic evidence. *Health & Place* 2010;**16**:175-190. doi: https://doi.org/10.1016/j.healthplace.2009.09.008

9. Saelens BE, Handy SL. Built Environment Correlates of Walking: A Review. *Medicine & Science in Sports & Exercise* 2008;**40**. doi: https://doi.org/10.1249/MSS.0b013e31817c67a4

10. Duncan MJ, Winkler E, Sugiyama T*, et al.* Relationships of Land Use Mix with Walking for Transport: Do Land Uses and Geographical Scale Matter? *Journal of Urban Health* 2010;**87**:782-795. doi: 10.1007/s11524-010-9488-7

11. Grasser G, Van Dyck D, Titze S, Stronegger W. Objectively measured walkability and active transport and weight-related outcomes in adults: a systematic review. *International Journal of Public Health* 2013;**58**:615-625. doi: 10.1007/s00038-012-0435-0

12. Daniels R, Mulley C. Explaining walking distance to public transport

The dominance of public transport supply. *Journal of Transport and Land Use* 2013;**6**:5-20. doi:

13. Gao J, Kamphuis CBM, Helbich M, Ettema D. What is ‘neighborhood walkability’? How the built environment differently correlates with walking for different purposes and with walking on weekdays and weekends. *Journal of Transport Geography* 2020;**88**:102860. doi: https://doi.org/10.1016/j.jtrangeo.2020.102860

14. van Soest D, R. TM, and Rogers CDF. Exploring the distances people walk to access public transport. *Transport Reviews* 2020;**40**:160-182. doi: 10.1080/01441647.2019.1575491

| **Supplementary Table 1. Summary of components included in the OBCT index: operationalisation, source, and year of data collection** | | | |
| --- | --- | --- | --- |
| **Component** | **Operationalisation** | **Source** | **Year** |
| **FEHI index** | Kernel density of all food retailers, weighted by the healthfulness of each food outlet type. | LOCATUS | 2006 |
| **Fast food density** | Kernel density, 1 km search radius | LOCATUS | 2006 |
| **Local food store density** | Kernel density, 1 km search radius | LOCATUS | 2006 |
| **Restaurant density** | Kernel density, 1 km search radius | LOCATUS | 2006 |
| **Supermarket density** | Kernel density, 1 km search radius | LOCATUS | 2006 |
| **Food delivery density** | Point density, 4 km circular radius | LOCATUS | 2006 |
| **Sports facility density** | Density of sport facilities per km2 per postcode 4 area. Only sport facilities that are present in the Real Estate Monitor 2015 database in each of the analysis years have been selected. These are: athletic tracks, indoor sport halls/ gyms, swimming pools (inside and outside), ice rinks, and artificial ski slopes. | Real Estate Monitor (ABF Research) with as original source the ‘BSvL – Nederlandse Sport Almanak NSA’ (data 2001–2007) and KNAU, Voetbalgids.com, skibanen Nederland, KNSB, and ZwembadGids (data 2010–2015) | 2006 |
| **Parking pressure** | Ratio between parking capacity and household car ownership | BGT, TOP10NL, BAG and RDW. | 2016 |
| **Highway distance** | Distance to the nearest A-/N-highway entrance/exit | Statistics Netherlands | 2016 |
| **Job travel time** | Time travel to the nearest 10,000 jobs | SPINlab, LISA | 2018 |
| **Land-use mix** | Entropy index of four main land-use classes: 1) industrial, commercial, public, military, and private; 2) residential; 3) urban green; and 4) sports and leisure | TOP10NL | 2006 |
| **Population density** | Number of inhabitants/km^2^ | Statistics Netherlands | 2006 |
| **Short public transport density** | Point density of short-range transports (buses, trams, metros, ferries) | NDOV of the Geographic service of the University of Groningen. | 2018 |
| **Distance to long public transport** | Distance to the nearest train station | NDOV of the Geographic service of the University of Groningen | 2018 |
| **Sidewalk density** | Density of sidewalks, pedestrian stairs, and residential areas suitable for walking | TOP10 NL via ESRI | 2008 |
| **Green space density** | Density of parks, forests, and graveyards | National Georegister | 2006 |
| **Intersection density** | Ratio of true intersections (>3 legs) and area size | TOP10 NL via ESRI | 2003 |
| **Density of retail and service destinations** | Area proportion devoted to two land use classes “commercial” and “socio-cultural services” | National Georegister | 2006 |
| **Bicycle pathway** | Percentage coverage of bicycle paths per neighborhood | TOP10 NL via ESRI | 2019 |
| LOCATUS is a commercial retail information provider. TOP10NL is the digital national topographic map. ESRI is the Environmental Systems Research Institute. NDOV is the national service for Dutch transit information by Foundation OpenGeo. SPINlab is the Free University Amsterdam’s Spatial Information Laboratory. LISA is National Information System of Workplaces. BAG (Basisregistratie Adressen en Gebouwen) is the Dutch national registry of addresses and buildings. BGT is the key register for large-scale topography of the Netherlands. RDW (Rijksdienst voor het Wegverkeer) is a Dutch organisation responsible for the registration and regulation of motor vehicles and driving licenses in the Netherlands. | | | |

| **Supplementary Table 2. ICD codes used for cardiovascular diseases** | |
| --- | --- |
|  | **Codes** |
| ICD-9 | 410, 4100, 41000, 41001, 41002, 4101, 41010, 41011, 41012, 4102, 41020, 41021, 41022, 4103, 41030, 41031, 41032, 4104, 41040, 41041, 41042, 4105, 41050, 41051, 41052, 4106, 41060, 41061, 41062, 4107, 41070, 41071, 41072, 4108, 41080, 41081, 41082, 4109, 41090, 41091, 41092, 411, 4110, 4111, 4118, 41181, 41189, 412, 413, 4130, 4131, 4139, 414, 4140, 41400, 41401, 41402, 41403, 41404, 41405, 41406, 41407, 4141, 41410, 41411, 41412, 41419, 4142, 4143, 4144, 4148, 4149, 428, 4280, 4281, 4282, 42820, 42821, 42822, 42823, 4283, 42830, 42831, 42832, 42833, 4284, 42840, 42841, 42842, 42843, 4289, 430, 431, 432, 4320, 4321, 4329, 433, 4330, 43300, 43301, 4331, 43310, 43311, 4332, 43320, 43321, 4333, 43330, 43331, 4338, 43380, 43381, 4339, 43390, 43391, 434, 4340, 43400, 43401, 4341, 43410, 43411, 4349, 43490, 43491, |
| ICD-10 | I20, I200, I201, I208, I209, I21, I210, I211, I212, I213, I214, I219, I22, I220, I221, I228, I229, I23, I230, I231, I232, I233, I234, I235, I236, I238, I24, I240, I241, I248, I249, I25, I250, I251, I252, I253, I254, I255, I256, I258, I259, I50, I500, I501, I509, I60, I600, I601, I602, I603, I604, I605, I606, I607, I608, I609, I61, I610, I611, I612, I613, I614, I615, I616, I618, I619, I62, I620, I621, I629, I63, I630, I631, I632, I633, I634, I635, I636, I638, I639, I64, I65, I650, I651, I652, I653, I658, I659, I66, I660, I661, I662, I663, I664, I668, I669, I67, I670, I671, I672, I673, I674, I675, I676, I677, I678, I679, I68, I680, I681, I682, I688, I69, I690, I691, I692, I693, I694, I698 |

| **Supplementary table 3. Population characteristics of the total study sample as well as stratified by individuals with and without missing data** | | | |
| --- | --- | --- | --- |
|  | **All** | **No missing data** | **Missing data** |
|  | 4,407,426 | 4,401,781 | 5,645 |
|  | |  |  |
| **Follow up (median [IQR])** | 14.0 [14.0; 14.0] | 14.0 [14.0; 14.0] | 14.0 [10.5; 14.0] |
| **Sex (%)** | |  |  |
| • Female | 51.4 | 51.4 | 50.3 |
| **Age at baseline (median [IQR])** | 56 [48; 65] | 56 [48; 66] | 55 [48; 62] |
| **Migration background (%)** | |  |  |
| • None | 91.2 | 91.2 | 71.3 |
| • Other Western | 4.4 | 4.4 | 19.9 |
| • Non-western | 4.5 | 4.4 | 8.8 |
| **Partner status (%)** | |  |  |
| • Single | 9.7 | 9.7 | 26.6 |
| • Widowed | 8.9 | 8.9 | 7.5 |
| • Separated | 8.2 | 8.2 | 15.4 |
| • Partner^1^ | 73.1 | 73.1 | 50.5 |
| **Annual household income in € (%)** | |  |  |
| • < 25% (< 16039) | 24.3 | 24.3 | 24.6 |
| • 25-75% (16039 to 27630) | 50.4 | 50.4 | 42.6 |
| • > 75% (> 27630) | 25.3 | 25.3 | 32.9 |
| **Area-level SES^2^ (mean (SD))** | 0.21 (0.81) | 0.21 (0.81) | 0.12 (0.88) |
| **Urbanisation (%)** | |  |  |
| • < 500 | 21.4 | 21.4 | 19.3 |
| • 500 to 1000 | 20.8 | 20.8 | 14.3 |
| • 1000 to 1500 | 20.1 | 20.1 | 21.0 |
| • 1500-2500 | 23.6 | 23.6 | 20.9 |
| • > 2500 | 14.1 | 14.1 | 19.3 |
| **PM_2.5_ in μg/m^3^ (median [IQR])** | 15.2 [14.0; 15.8] | 15.2 [14.0; 15.8] | 14.9 [13.9; 15.8] |
| 1 Partner included both marriage and registered partnership  2 Socio-economic status | | | |


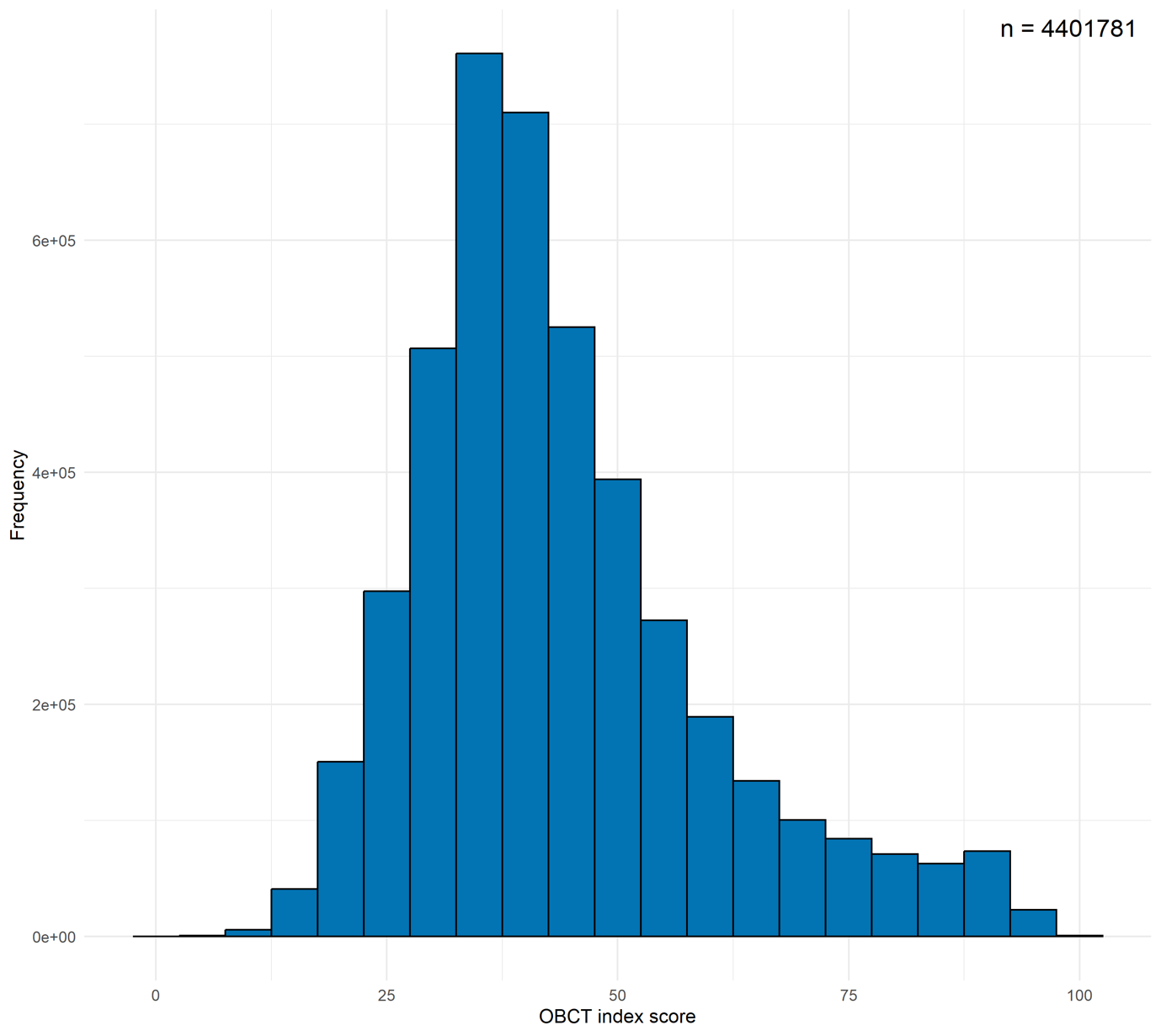


**Supplementary figure 1.** **Distribution of the OBCT index score at baseline among individuals included in the study.**

Mean ± SD = 55.5 ± 17.2


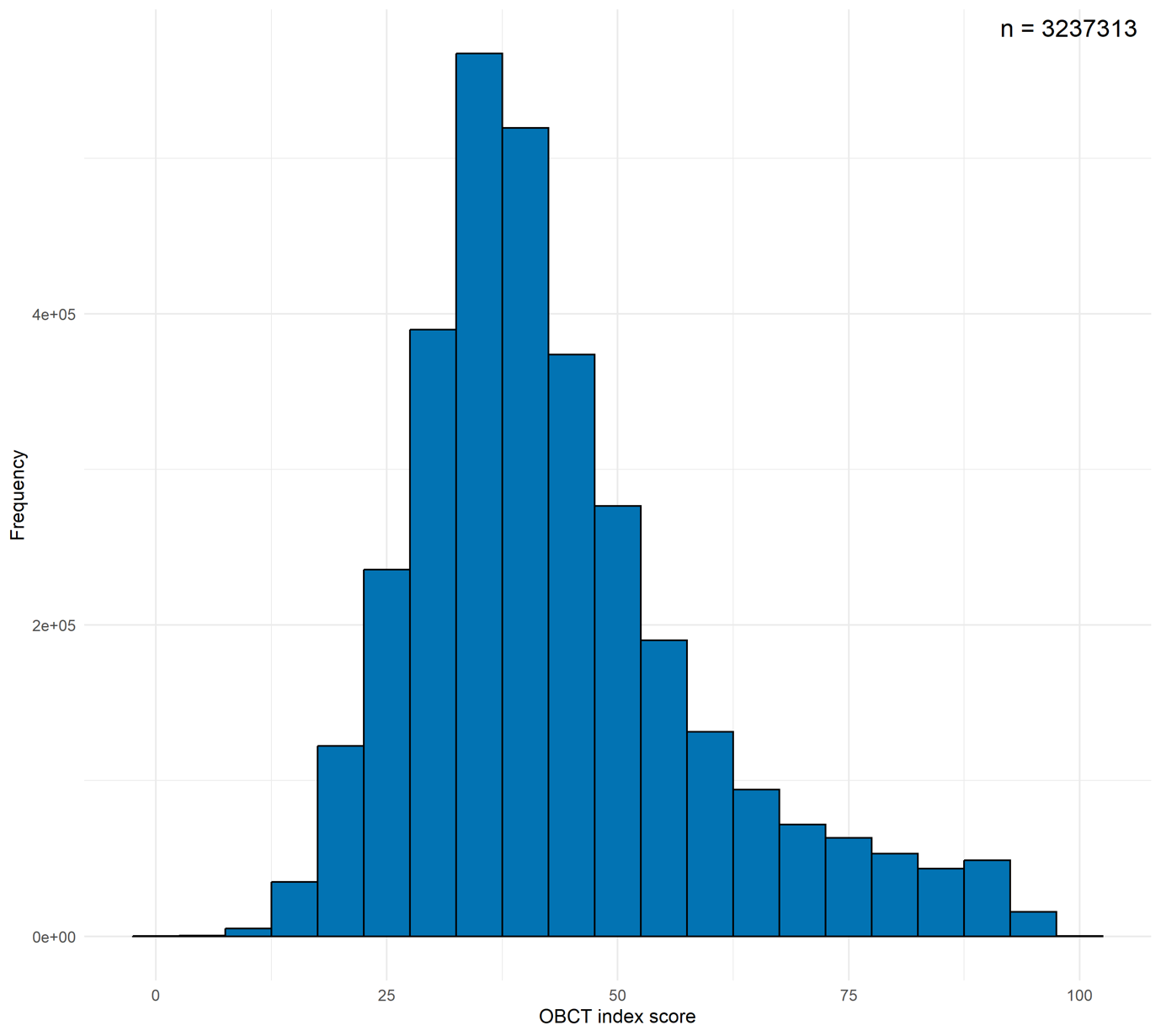


**Supplementary figure 2.** **Distribution of the OBCT index score at baseline among individuals excluded from the study because they moved to a different address within the Netherlands during follow-up.**

Mean ± SD = 54.0 ± 17.2


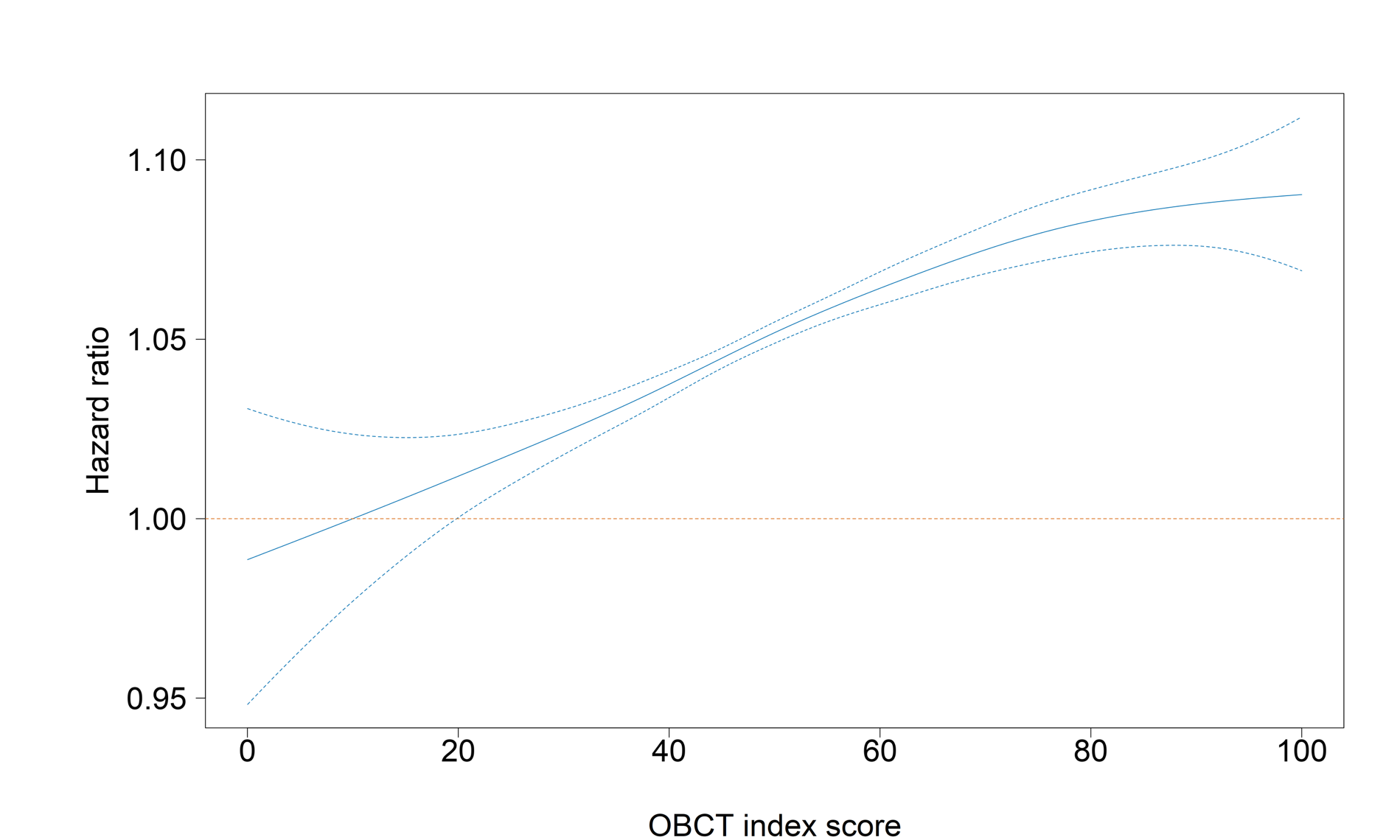


# **Supplementary figure 3. Associations between the OBCT index score using only the components available at or near baseline, and any CVD using penalized spline regression (df = 3).**

Models adjusted for age, sex, migration background, partner status, household income, comorbidities, area level SES, PM_2_._5_ exposure, and urbanicity.

* Remained statistically significant after false very rate correction for multiple testing.

An OBCT index score of 10 is set as reference value.


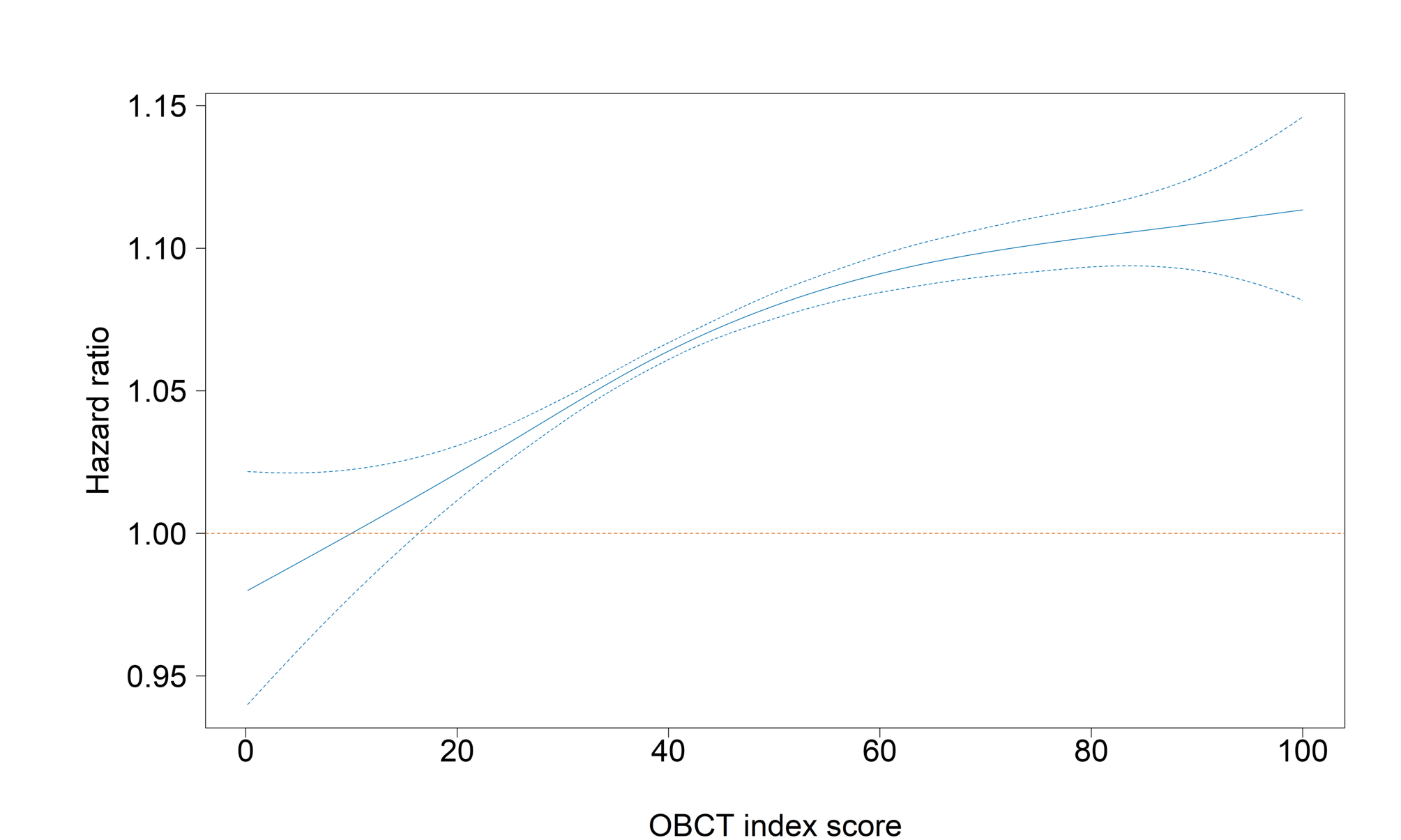


# **Supplementary figure 4. Associations between the full OBCT index score and any CVD using penalized spline regression (df = 3).**

Models adjusted for age, sex, migration background, partner status, household income, comorbidities, area level SES, PM_2_._5_ exposure, and urbanicity.

* Remained statistically significant after false very rate correction for multiple testing.

An OBCT index score of 10 is set as reference value.


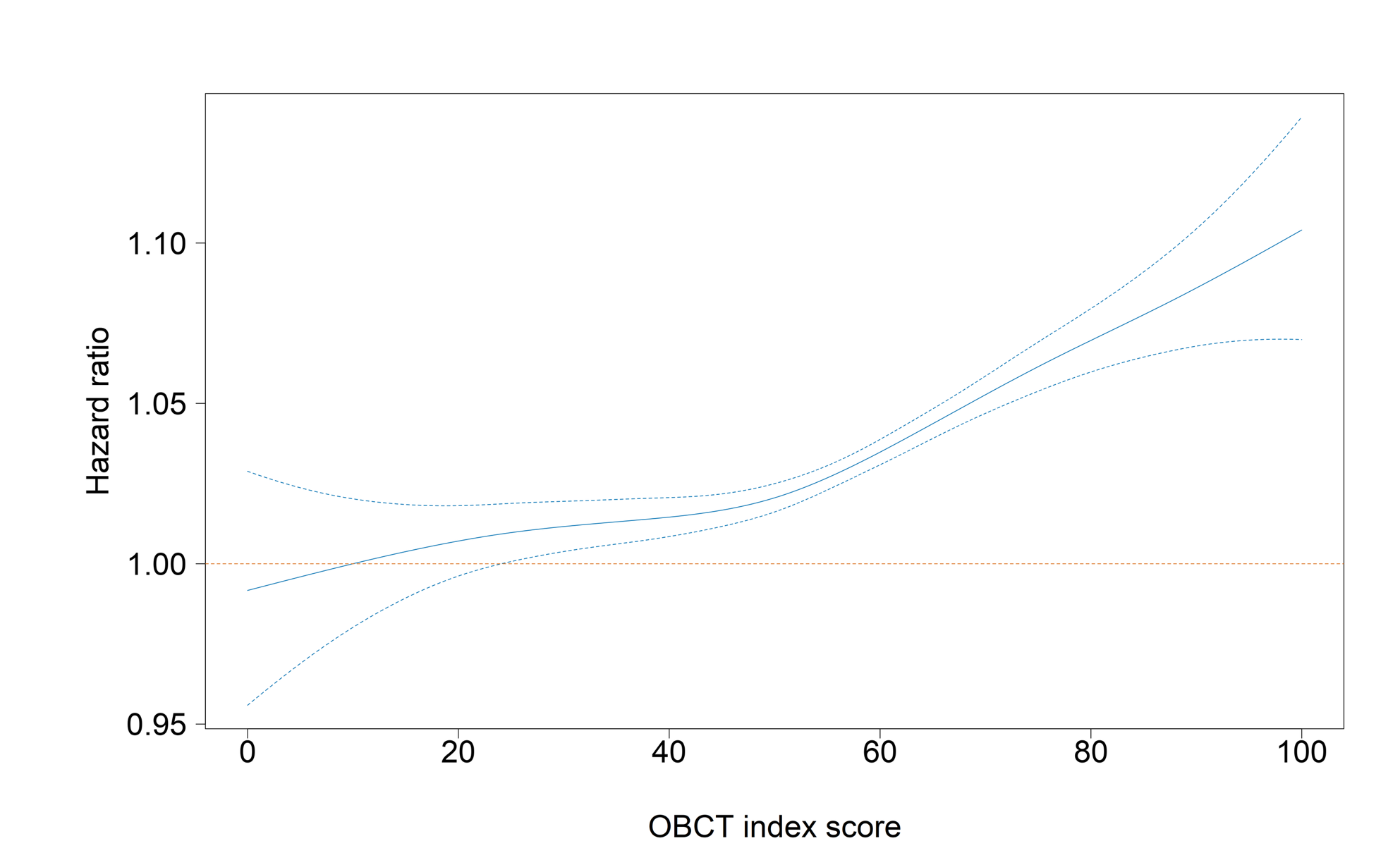


# **Supplementary figure 5. Associations between the full OBCT index score using the densities of various food retailers instead of the FEHI, and any CVD using penalized spline regression (df = 3).**

Models adjusted for age, sex, migration background, partner status, household income, comorbidities, area level SES, PM_2_._5_ exposure, and urbanicity.

* Remained statistically significant after false very rate correction for multiple testing.

An OBCT index score of 10 is set as reference value.


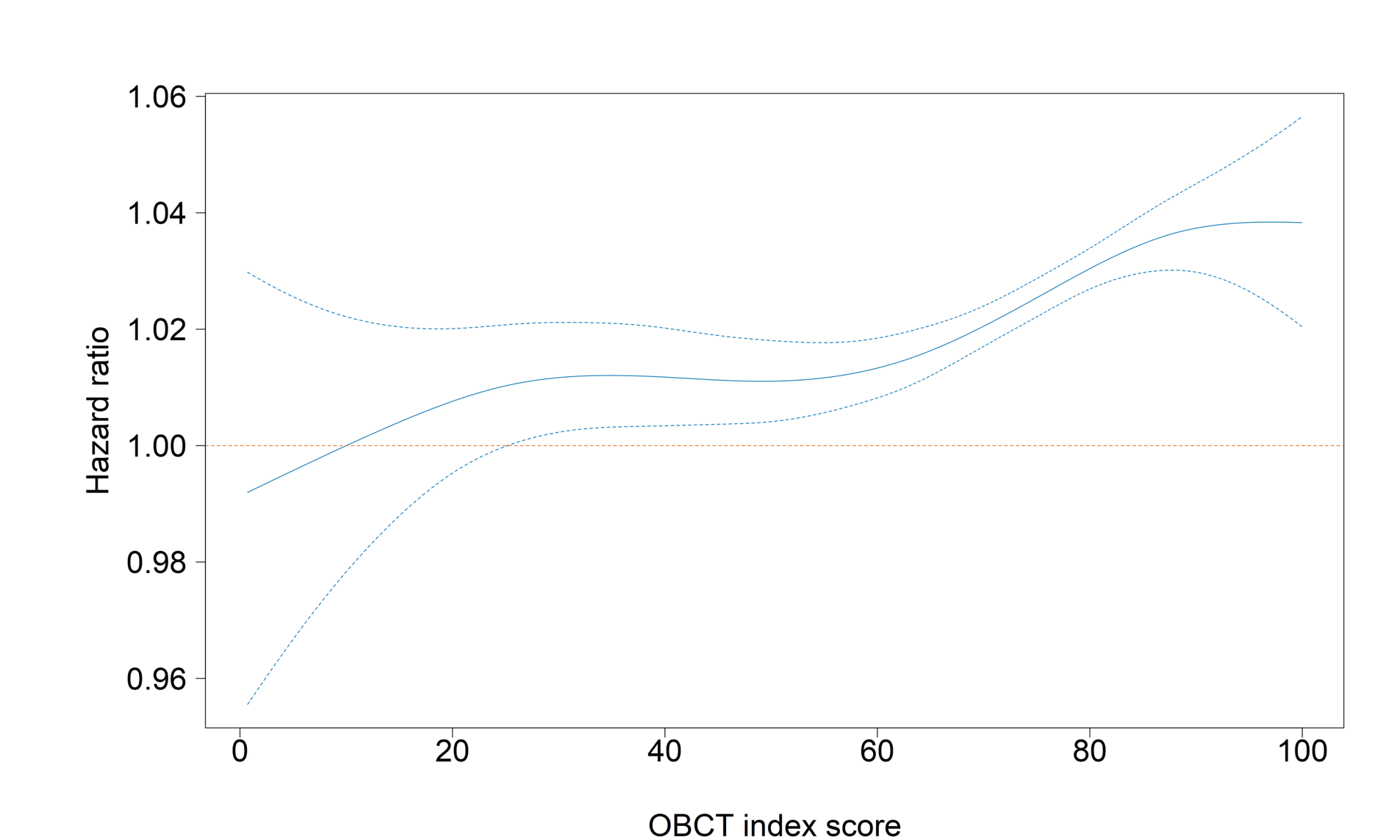


**Supplementary figure 6. Associations between the full OBCT index score using only the components available at or near baseline, and the densities of various food retailers instead of the FEHI, and any CVD using penalized spline regression (df = 3).**

Models adjusted for age, sex, migration background, partner status, household income, comorbidities, area level SES, PM_2_._5_ exposure, and urbanicity.

* Remained statistically significant after false very rate correction for multiple testing.

An OBCT index score of 10 is set as reference value.


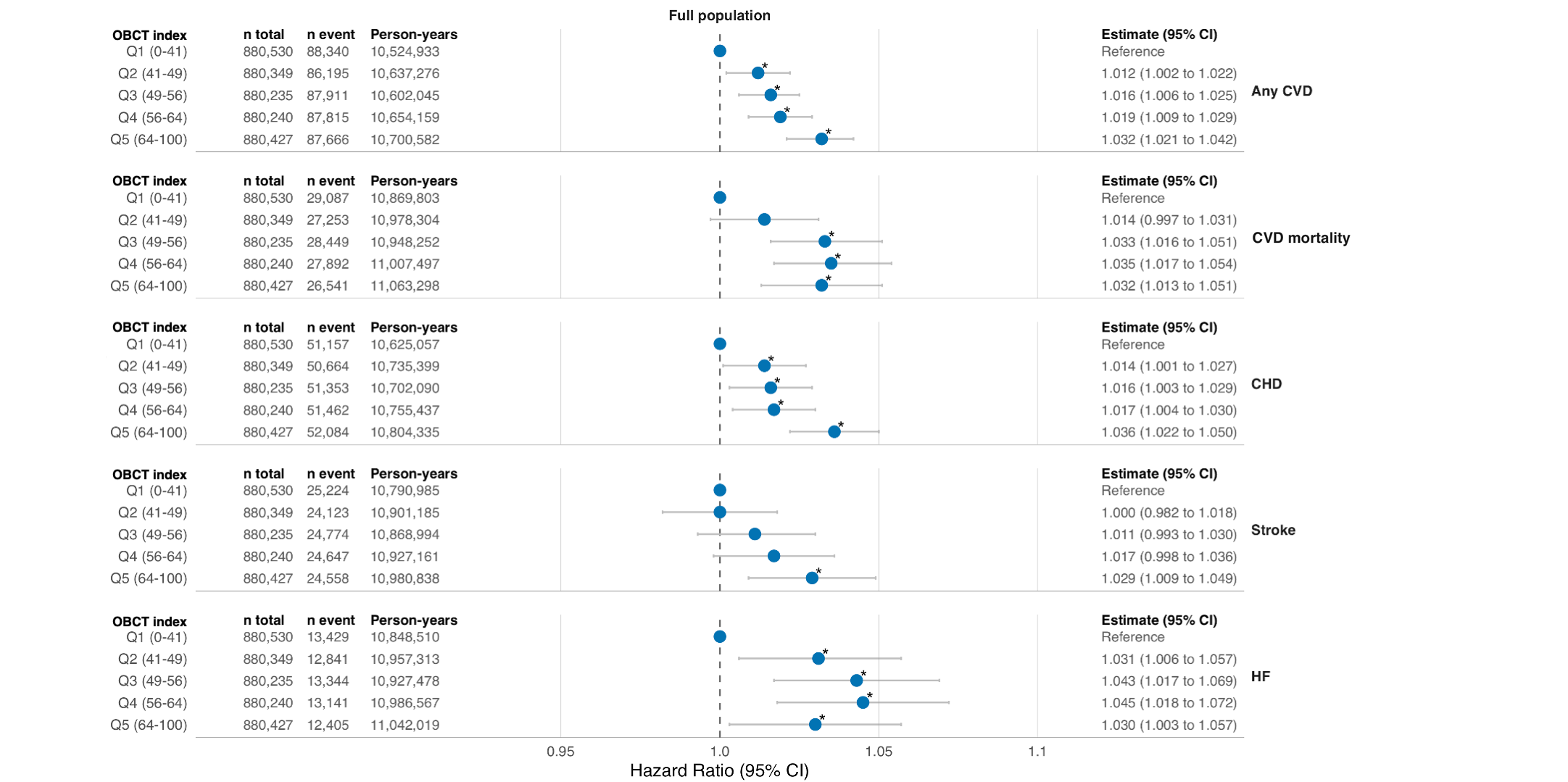


# **Supplementary figure 7. Associations between the Obesogenic Built-environmental CharacterisTics (OBCT) index in a 500m circular buffer score and cardiovascular outcomes.**

Models adjusted for age, sex, migration background, partner status, household income, comorbidities, area level SES, PM_2_._5_ exposure, and urbanicity.

* Remained statistically significant after false very rate correction for multiple testing.

CVD, cardiovascular disease; CHD, coronary heart disease; HF, heart failure.


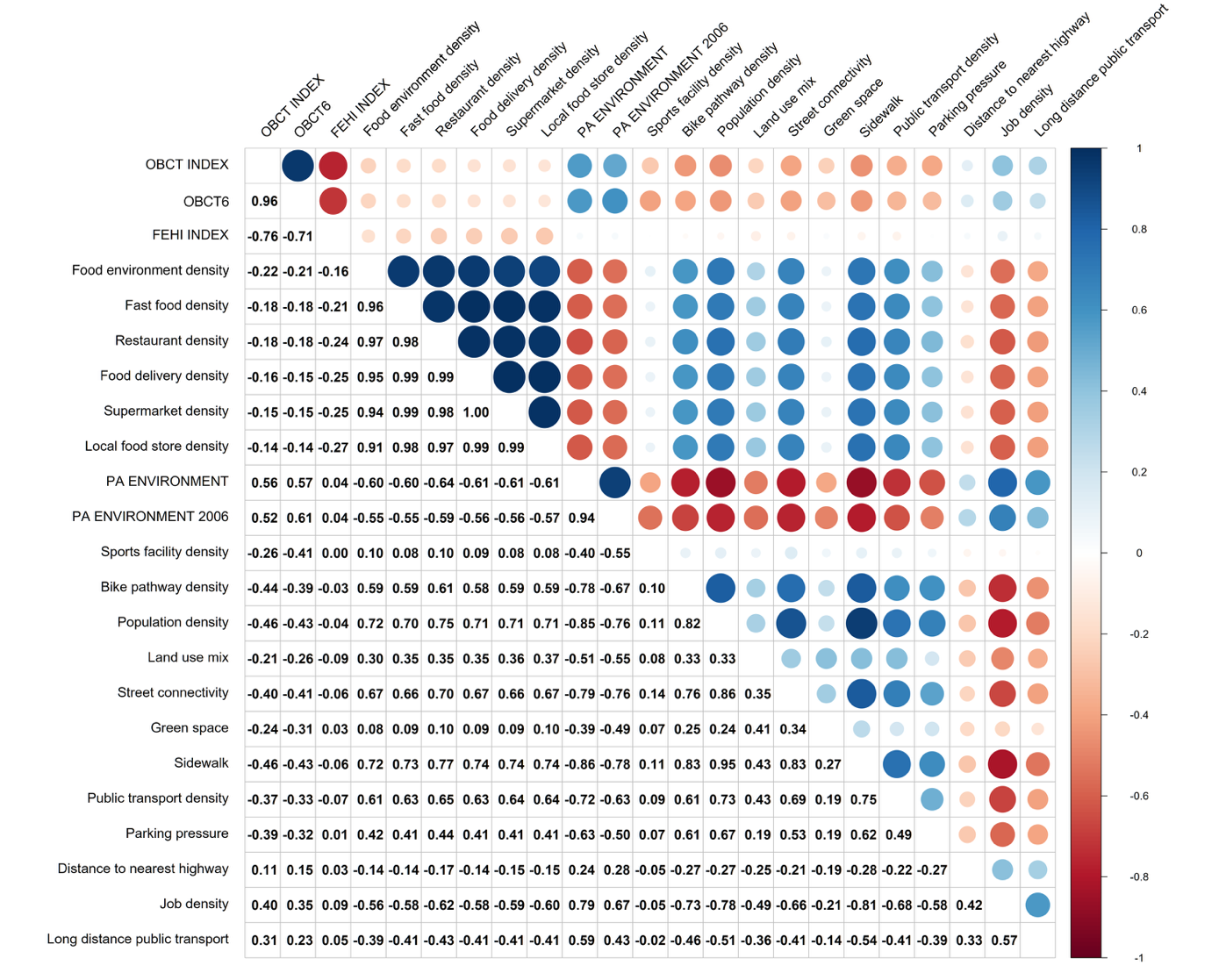


**Supplementary figure 8.** **Spearman correlation matrix of the OBCT index constructs and components.**

Empty cells denote nonsignificant correlation (p > 0.05).

OBCT6 = index using only the components available at or near baseline.

FEHI: Food Environment Healthiness Index; PA: physical activity.
